# Increased Variability of Genomic Transcription in Schizophrenia

**DOI:** 10.1038/srep17995

**Published:** 2015-12-10

**Authors:** Fuquan Zhang, Yin Yao Shugart, Weihua Yue, Zaohuo Cheng, Guoqiang Wang, Zhenhe Zhou, Chunhui Jin, Jianmin Yuan, Sha Liu, Yong Xu

**Affiliations:** 1 Wuxi Mental Health Center, Nanjing Medical University, Wuxi, Jiangsu Province, China; 2 Unit on Statistical Genomics, Division of Intramural Research Program, National Institute of Mental Health, National Institutes of Health, Bethesda, Maryland, United States of America; 3 Institute of Mental Health, Sixth Hospital, Peking University; Beijing 100191, China; 4 Key Laboratory of Mental Health, Ministry of Health & National Clinical Research Center for Mental Disorders (Peking University), Beijing, 100191, China; 5 Department of Psychiatry, First Hospital /First Clinical Medical College of Shanxi Medical University, Taiyuan, China

## Abstract

Schizophrenia (SZ) is a severe chronic mental disorder with a high heritability. Current microarray analyses typically focus on identifying differentially expressed genes or enriched pathways relevant to phenotypes. Whether there is a variability change of the genomic transcription in diseases has rarely been explored. In this study, we compared coefficient of variation (CV, the ratio of the standard deviation to the mean) of genome transcription of early-onset SZ (EOS) patients with controls in a blood mRNA microarray dataset and a blood microRNA (miRNA) microarray dataset. Furthermore, we compared CV of the expression levels of 17 genes in blood of the 30 patients before and after the 12-week treatment using real-time quantitative PCR (RT-qPCR) analysis. Our results indicated a significant increase of CV of genome transcription in patients compared with controls in both the mRNA and the miRNA datasets. The 30 after-treatment patients showed a significant decrease of CV of gene expression compared with the before-treatment patients. Our study may implicate the blood gene expression variability in SZ, providing further evidence supporting the abnormality of peripheral blood transcriptome in SZ. Given that peripheral blood can be easily collected from patients and followed longitudinally, our results may indicate a new way to facilitate the identification of the signatures of clinical subtypes, their prognosis and treatment response.

Schizophrenia (SZ) is a severe chronic mental disorder with a high heritability. It profoundly disrupts such key traits of human cognition and personality. SZ patients tend to first present with overt symptoms during late adolescence or early adulthood. When the disease manifests before age 18, it is categorized as early-onset SZ (EOS), a subcategory of SZ associated with more familial vulnerability and poor outcomes^1^.

It has been hypothesized that the gene expression is the most fundamental level at which the genotypes critically influence the SZ phenotypes. Peripheral blood mononuclear cells (PBMCs) can be easily collected from patients and followed longitudinally with gene expression analyses, providing a way of identifying the signatures of clinical subtypes, their prognosis and treatment response.

Microarray-based gene expression profiling has been used to explore peripheral molecular disruption in SZ patients. Current microarray analyses typically focus on identifying differentially expressed genes or enriched pathways in different phenotypes. Whether there is a variability change of the genomic transcription in diseases has rarely been explored. In this study, we aim to investigate the possible change of genome expression variability in SZ. We used the coefficient of variation (CV) as a measure to evaluate the magnitude of genome expression variability. CV, the ratio of the standard deviation, is a standardized measure of dispersion of a probability distribution or frequency distribution.

## Results

**Figure 1:**
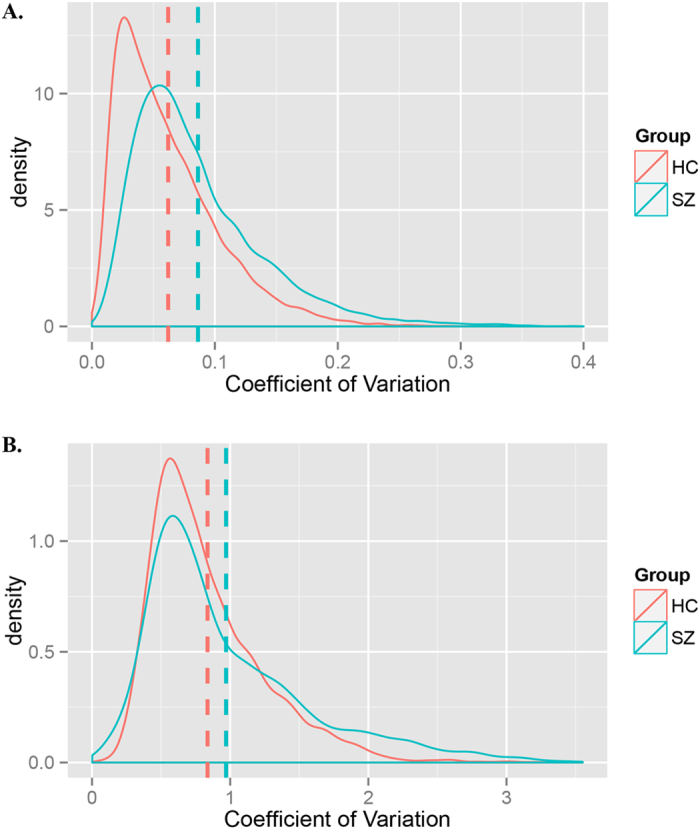
Density curves of expression CV in SZ patients and controls. The vertical dashed lines indicate mean values. (**A**) Density curves of mRNA expression CV in SZ patients and controls. (**B**) Density curves of miRNA expression CV in SZ patients and controls.

We compared the CV of genome transcription of SZ patients with controls in two microarray datasets. In the mRNA dataset, there was a 38.71% increase of CV of genome transcription in patients compared with controls 19.75% (P < 1.00E-30); while in the mRNA dataset, there was a 19.75% increase of CV of miRNA expression in the patients compared with controls (P = 3.30E-30, Table 1, Fig. 1).

We compared CV of expression levels of the 17 genes in the patients before and after the treatment using RT-qPCR analysis. After the treatment, the 30 patients showed a significant decrease of CV of expression levels of the 17 genes (16.05%, P = 0.015, Table 2). However, there were no significant changes of expression levels for each of the 17 genes after the treatment (Table 3).

## Discussion

The transcriptome sits between environmental influence and the genetic susceptibility to SZ and thus may serve as a bridge between certain endophenotypes and the genetic changes that lead to the disorder. Unlike the most of the previous expression studies, we focus on alterations of expression variability in SZ patients.

Changes of gene expression variability have been observed in different biological conditions or phenotypes^2^,^3^. Our findings support the increased genome expression variability in drug-free SZ patients. Strikingly, the magnitude of the changes was relative large, with a nearly 40% and 20% increase of CV in mRNA and miRNA profiles, respectively. The increased CV may reveal a more irregular expression pattern in SZ compared with controls, plausibly resulting from the stochastic deregulation of the genome expression^4^,^5^.

miRNAs are a class of vital gene expression regulators posttranscriptionally repress expression of target genes by mRNA degradation or translational inhibition. Our miRNA microarray dataset indicated a down-regulation and an increased variance of expression levels in SZ patients, both of which may contribute to genome dysregulation.

Interestingly, although none of the 17 genes showed significant changes after the treatment, collectively, their expression variances significantly decreased after the treatment, consistent with the hypothesis that biologically relevant genes can have differential variability without differential expression^6^. The reduction of CV after the treatment further supports the relevance of CV of gene expression in SZ. To the best of our knowledge, this is the first time that trait- and treatment-related CV changes of transcripts in SZ patients were reported.

The limitation of our study might be the relative small sample size in the two microarray datasets. However, one can argue that the large number of features (genes or miRNAs) and the employing of multiple datasets may compensate this limitation. A caveat in mind was that our results were derived from peripheral blood; thus, due care should be taken when extrapolating these results into the brain of SZ patients. The strength of our study was the enrollment of drug-free patients and the relative good match of the two groups of subjects in gender and age. We are aware of the fact that expression variability may result from other sources, such as some level of heterogeneity related to sample collection. However, it is interesting to see that our experiments revealed the striking differential variability in the patients with the consistency across all the datasets.

In summary, our study indicated the increase of gene expression variability in blood of SZ patients, providing evidence supporting the alteration of peripheral blood transcriptome in the disease.

## Methods

### Subjects

All participants were unrelated Han Chinese recruited from the north of China. Consensus diagnoses were made by at least two experienced psychiatrists independently according to the Diagnosis and Statistical Manual of Mental Disorders Fourth Edition (DSM-IV) criteria for SZ. Patients with unanimous diagnosis were enrolled into the study.

For the real-time RT-qPCR analysis, we enrolled 38 SZ patients who were drug-free for at least one month before the enrollment; among them 30 patients (14 males and 16 females, aged 34.5 ± 11.0 years) were successfully followed-up with a 12-week period of antipsychotic treatment (see Supplementary File for detailed information). The clinical effects were assessed by trained and experienced psychiatrists with the Positive and Negative Syndrome Scale (PANSS) respectively before and after 12-week treatment. All patients showed clinical improvement according to the PANSS reductive ratio more than 25%. All patients participating in the study were treated with one of the oral second generation or atypical antipsychotics (SGA) and tracked for 12-week continuous medication after baseline assessments. A total of 48 healthy controls (17 males and 31 females, aged 31.6 ± 6.88 years) were recruited from local communities or were undergoing routine health check-ups. Subjects with relevant physical diseases or a history of major psychiatric disorders or suicidal behavior were excluded, and those who had a first-degree relative with a history of severe mental disorder or suicidal behavior were also excluded.

There was no significant difference in gender or age between SZ cases and controls in the two cohorts of samples. Total RNA was isolated from peripheral blood mononuclear cells (PBMCs) using TRIzol (Invitrogen; USA) with on-column DNase I treatment as described by the manufacturer. Blood samples of each participant were collected on early morning before breakfast. Following the diagnosis, blood samples of the schizophrenia patients were collected next morning.

The study was approved by Medical Research Ethics Committee of Shanxi Medical University, and all experiments were performed in accordance with the approved guidelines and regulations. Informed consent was signed by both the teenage participants and their parents or care takers.

### The mRNA microarray dataset

The blood-based mRNA microarray dataset involves 17199 probes with valid values from 18 first-onset SZ patients (8 males and 10 females, aged 14.78 ± 1.70 years ranging from 10–18 years) and 12 healthy controls (6 males and 6 females, aged 14.75 ± 2.14 years ranging from 10–17 years) (NCBI GEO: GSE54913, see Supplementary File for detailed information on data processing).

### The miRNA microarray dataset

The blood-based miRNA microarray dataset involves 2967 non-missing probes from 15 first-onset SZ patients (8 males and 7 females, aged 13.80 ± 1.93 years ranging from 12–17 years) and 15 healthy controls (7 males and 8 females, aged 14.07 ± 1.82 years ranging from 9–16 years) (NCBI GEO: GSE54578, see Supplementary File for detailed information on data processing)^7^.

### RT-qPCR analysis

Total RNA was isolated from peripheral blood mononuclear cells (PBMCs) using TRIzol (Invitrogen; USA) with on-column DNase I treatment as described by the manufacturer. cDNA was synthesized using High Capacity RNA-to-cDNA Kit (Invitrogen; USA) as described by the manufacturer. The primers were listed in Supplementary Table S2. PCR was performed using a ViiA 7 Real-time PCR System (Applied Biosystems) for 10 min at 95 °C, and then 40 cycles consisting of 10 s at 95 °C, 60 s at 60 °C, 15 s at 95 °C, followed by a subsequent standard dissociation protocol to ensure that each amplicon was a single product. All quantifications were normalized to GAPDH.

### Statistical analysis

R^8^ was used to perform the data processing and analyses. Density curves were plotted with the R package ggplot2^9^. For RT-qPCR analysis, the comparative Ct (2^−ΔΔCT^) method was used for the quantification of transcripts. The differences of CV between two groups were analyzed using paired Wilcoxon rank-sum test.

## Additional Information

**How to cite this article**: Zhang, F. *et al.* Increased Variability of Genomic Transcription in Schizophrenia. *Sci. Rep.* **5**, 17995; doi: 10.1038/srep17995 (2015).

## Supplementary Material

Supplementary Information

## Figures and Tables

**Table 1:** Coefficient of variation of transcriptions in SZ patients and controls.

Platform	Features	Samples	Control	SZ	Change rate	P
Microarray	17199 mRNA probes	18 SZ/12 controls	0.062 ± 0.043	0.086 ± 0.054	38.71%	<100E-30
Microarray	2967 miRNA probes	15 SZ/15 controls	0.81 ± 0.41	0.97 ± 0.60	19.75%	330E-30

**Table 2:** Coefficient of variation of transcriptions in SZ patients before and after the treatment.

Platform	Features	Samples	SZ_0W	SZ_12W	Change rate	P
RT-qPCR	17 mRNAs	30 SZ patients	0.81 ± 0.52	0.68 ± 0.47	−16.05%	0.015

SZ_0W: SZ patients before treatment; SZ_12W: SZ patients after 12-week treatment.

**Table 3:** Expression levels of in the 30 SZ patients before and after the treatment.

Gene	SZ (n = 30)	SZ_12w (n = 30)	FC	P	FDR
AKT1	0.076 ± 0.022	0.071 ± 0.017	1.00	0.62	0.81
BRCA1	6.57E-3 ± 2.79E-3	5.43E-3 ± 1.85E-3	1.00	0.13	0.38
CCDC134	0.012 ± 0.026	2.11E-3 ± 2.95E-3	0.99	0.013	0.11
FAHH	4.84E-3 ± 2.25E-3	5.45E-3 ± 2.45E-3	1.00	0.21	0.44
FOS	1.09 ± 0.55	1.29 ± 0.84	1.38	0.47	0.66
HTR4	1.70E-4 ± 9.44E-5	1.52E-4 ± 8.85E-5	1.00	0.45	0.66
JUN	4.49E-3 ± 3.60E-3	3.83E-3 ± 2.04E-3	1.00	0.92	0.98
MYOD1	2.22E-3 ± 3.00E-3	1.00E-3 ± 1.87E-3	1.00	0.011	0.11
NKAPL	3.40E-3 ± 2.20E-3	2.45E-3 ± 1.12E-3	1.00	0.085	0.29
RGS2	0.75 ± 0.43	0.69 ± 0.28	0.93	0.98	0.98
STAT3	0.049 ± 0.031	0.053 ± 0.027	1.00	0.25	0.48
UBD	3.82E-3 ± 4.70E-3	1.68E-3 ± 1.63E-3	1.00	0.033	0.14
VDR	0.010 ± 4.81E-3	8.95E-3 ± 1.97E-3	1.00	0.73	0.89
ZIC2	3.97E-3 ± 6.36E-3	8.78E-4 ± 1.27E-3	1.00	0.027	0.14
EGR1	0.19 ± 0.21	0.20 ± 0.15	1.00	0.19	0.44
SKIL	5.28E-3 ± 2.02E-3	5.57E-3 ± 1.70E-3	1.00	0.39	0.66
NFKB	0.026 ± 0.012	0.026 ± 9.44E-3	1.00	0.97	0.98

FC: fold change.
