# Supplementary material for: Increased Variability of Genomic Transcription in Schizophrenia
Source: Sci Rep. 2015 Dec 10;5:17995. doi: 10.1038/srep17995 (PMC4675071; doi:10.1038/srep17995)
Supplement: Supplementary Information [file srep17995-s1.doc]

SUPPLEMENTARY FILE

**Table of Contents**

**1. Introduction**

**2. Clinical medication and assessments**

**3. mRNA expression data quality assessment**

3.1. Array Information

3.2. RNA extraction

3.3. Raw mRNA data normalization and low intensity filtering

3.4. Quality assessment of mRNA data after filtering

**4. microRNA expression data quality assessment**

4.1. Array Information

4.2. RNA quality

4.3. Raw mRNA data normalization and low intensity filtering

4.4. Quality assessment of mRNA data after filtering

**5. Analysis of gene expression by RT-qPCR**

**6. Supplementary tables**

6.1. Supplementary Table S1. Primers of mRNAs in the RT-qPCR analysis

**1. Introduction**

This file contains detailed information about Clinical medication and assessments of the schizophrenia patients (section 2), preprocessing of the mRNA microarray dataset (section 3), preprocessing of the microRNA microarray dataset (section 4), RT-qPCR method (sections 5), and supplementary tables (section 6).

**2. Clinical medication and assessments**

Consensus diagnoses were made by at least two experienced psychiatrists independently according to the Diagnosis and Statistical Manual of Mental Disorders Fourth Edition (DSM-IV) criteria for SZ. Patients with unanimous diagnosis were enrolled into the study.

The clinical effects were assessed by trained and experienced psychiatrists with the Positive and Negative Syndrome Scale (PANSS) respectively before and after 12-week treatment. All patients got clinical improvement according to the PANSS reductive ratio more than 25%.

All patients participating in the study were treated with one of the oral second generation or atypical antipsychotics (SGA) and tracked for 12-week continuous medication after baseline assessments. Among the 30 patients, 16 were treated with risperidone (average dosage 4.5 mg, ranging from 2-6 mg); 7 with olanzapine (average dosage 13 mg, ranging from 5-20 mg); 3 with quetiapine (average dosage 550 mg, ranging from 100-800mg); 2 with aripiprazole (average dosage 12.5 mg, ranging from 15-30 mg) and 2 with ziprasidone (average dosage 135 mg, ranging from 40-140 mg). All patients were given one of the five SGAs once daily, from an initial dose and increasing to a curative dose for the next 2-4 weeks. The specific dose (maintenance dose, and increases in dosage) was adjusted according to side effects and clinical assessment. If necessary, small doses of benzodiazepines were prescribed for agitation.

**3. mRNA expression data quality assessment**

**3.1. Array Information**

Arraystar Human 8x60K LncRNA Microarray v2.0 is designed for the global profiling of human LncRNAs and protein-coding transcripts. 33,045 LncRNAs and 30,215 coding transcripts can be detected by our second-generation LncRNA microarray. Each transcript is represented by a specific exon or splice junction probe which can identify individual transcript accurately. Positive probes for housekeeping genes and negative probes are also printed onto the array for hybridization quality control.

**3.2. RNA quality**

The NanoDrop ND-1000 was used for accurate for measurement of concentrations (OD260), protein contamination (ratio OD260/OD280) and organic compounds contamination (ratio OD260/OD230). The integrity of RNA can be assessed by electrophoresis on a denaturing agarose gel. Intact total RNA run on a denaturing gel will have sharp 28S and 18S rRNA bands (eukaryotic samples). The 28S rRNA band are approximately twice as intense as the 18S rRNA band.

**3.2. Raw mRNA data normalization and low intensity filtering**

Quantile normalization and subsequent data processing were performed using the GeneSpring GX v11.5.1 software package (Agilent Technologies). After quantile normalization of the raw data, mRNAs that at least 20 out of 30 samples have flags in Present or Marginal (“All Targets Value – mRNAs”) were chosen for differentially expressed mRNAs screening.

**3.3. Quality assessment of mRNA data after filtering**

The Box Plot is a convenient way to quickly visualize the distributions of a dataset. It is commonly used for comparing the distributions of the intensities from all samples. After normalization, the distributions of log2-ratios among all samples are nearly the same.


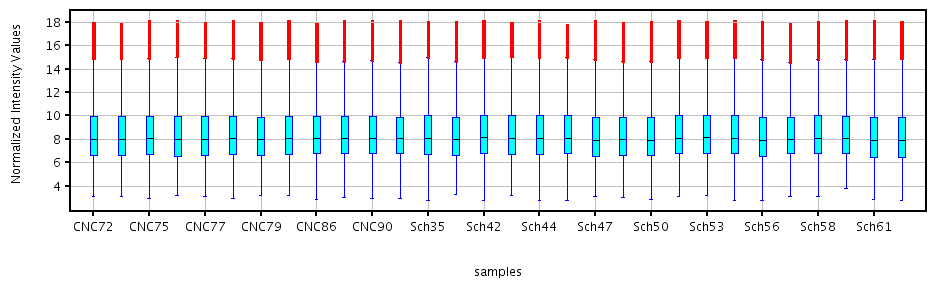


The Scatter-Plot is a visualization method used for assessing the mRNA expression variation (or reproducibility) between the two compared groups of samples or two compared samples. The values of X and Y axes in the Scatter-Plot are the normalized signal values of two samples (log2 scaled) or the averaged normalized signal values of groups of samples (log2 scaled). The green lines are Fold Change Lines (The default fold change value given is 2.0). The mRNAs above the top green line and below the bottom green line indicated more than 2.0 fold change of mRNAs between the two compared samples or the two compared groups of samples.

The following Scatter-Plot is for the schizophrenia patients group (T1) vs the control group (C).


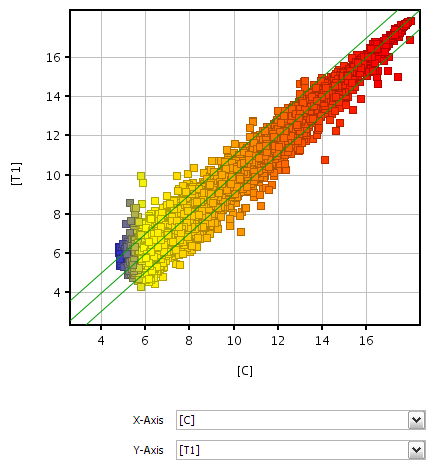


**4. microRNA expression data quality assessment**

**4.1. Array Information**

The 7th generation of miRCURY^TM^ LNA Array (v.18.0) (Exiqon) contains 3100 capture probes, covering all human, mouse and rat microRNAs annotated in miRBase 18.0, as well as all viral microRNAs related to these species. In addition, this array contains capture probes for 25 miRPlus™ human microRNAs.

**4.2. RNA extraction**

Total RNA was isolated using TRIzol (Invitrogen) and miRNeasy mini kit (QIAGEN) according to manufacturer’s instructions, which efficiently recovered all RNA species, including miRNAs. RNA quality and quantity was measured by using nanodrop spectrophotometer (ND-1000, Nanodrop Technologies) and RNA Integrity was determined by gel electrophoresis.

**4.3. Raw miRNA data normalization and low intensity filtering**

Scanned images were then imported into GenePix Pro 6.0 software (Axon) for grid alignment and data extraction. Replicated miRNAs were averaged and miRNAs that intensities>=50 in all samples were chosen for calculating Median normalization factor. Expressed miRNA data were normalized using the Median normalization and chosen for differentially expressed miRNAs screening.

**4.4. Quality assessment of mRNA data after filtering**

After normalization, the distributions of log2-ratios across every sample are nearly the same.


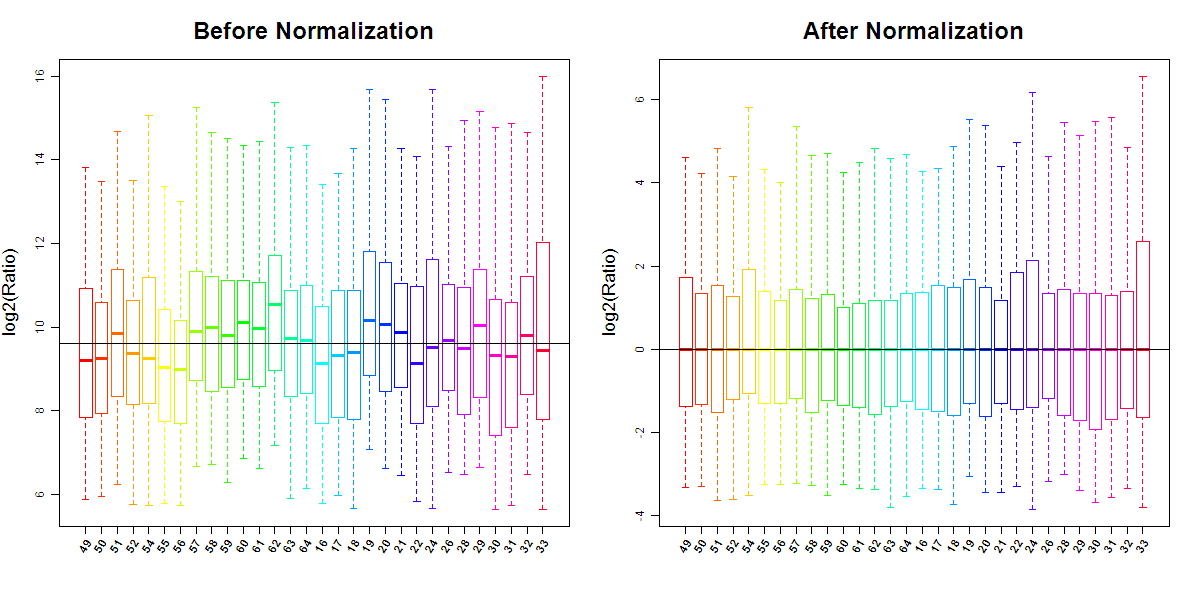


Box Plot of miRNA data after filtering. Left: non-normalized log2-ratio data; right: Median normalized log2-ratio data.

**5. Analysis of gene expression by RT-qPCR**

Total RNA was isolated from peripheral blood mononuclear cells (PBMCs) using TRIzol (Invitrogen; USA) with on-column DNase I treatment as described by the manufacturer. cDNA was synthesized using High Capacity RNA-to-cDNA Kit (Invitrogen; USA) as described by the manufacturer. The primers were listed in Supplementary Table S7. PCR was performed using a ViiA 7 Real-time PCR System (Applied Biosystems) for 10 min at 95 °C, and then 40 cycles consisting of 10 s at 95 °C, 60 s at 60 °C, 15 s at 95 °C, followed by a subsequent standard dissociation protocol to ensure that each amplicon was a single product. All quantifications were normalized to GAPDH.

**6. Supplementary tables**

**Supplementary Table S1 |** Primers of mRNAs in the RT-qPCR analysis

| Genes | Primers |
| --- | --- |
| GAPDH | F: 5’GGGAAACTGTGGCGTGAT3’ |
|  | R: 5’GAGTGGGTGTCGCTGTTGA3’ |
| AKT1 | F: 5’CACACCACCTGACCAAGATG3’ |
|  | R: 5’GCACCCGAGAAATAAAAACC3’ |
| BRCA1 | F: 5’CACCAAGGTCCAAAGCGA3’ |
|  | R: 5’TTGCATGGAAGCCATTGTC3’ |
| CCDC134 | F: 5’AAGAGAAACGCCGAAAGAA3’ |
|  | R: 5’GGCCCCTGAGAGAAAATCC3’ |
| FAAH | F: 5’CTTCAAAGGTGATTTCGTGGAC3’ |
|  | R: 5’TTTCCAGCCGAACGAGACTT3’ |
| FOS | F: 5’TTTGCCTAACCGCCACGAT3’ |
|  | R: 5’CTGCGGGTGAGTGGTAGTAAGAG3’ |
| HTR4 | F: 5’TCGACGGTTATCCTGATGGC3’ |
|  | R: 5’CAGCAGATCCGCAAAAGCAA3’ |
| JUN | F: 5’GACCTTATGGCTACAGTAACC3’ |
|  | R: 5’TGCTGGACTGGATTATCAGG3’ |
| MYOD1 | F: 5’GCCACAACGGACGACTTCT3’ |
|  | R: 5’CGAGTGCTCTTCGGGTTTC3’ |
| NKAPL | F: 5’GCTCTTTACCCCTTTAGTCG3’ |
|  | R: 5’ATGGCTCTCTTCCTTCTCGT3’ |
| RGS2 | F: 5’TCCAGCGGGAGAACGATAAT3’ |
|  | R: 5’GCTCAAACGGGTCTTCCAATC3’ |
| STAT3 | F: 5’TGGAAATAATGGTGAAGGTGC3’ |
|  | R: 5’ATCTGGGGTTTGGCTGTGT3’ |
| UBD | F: 5’CAAAGAGAAGACCATCCACCT3’ |
|  | R: 5’CATCACCTGACTCCACAAGAA3’ |
| VDR | F: 5’GGGTTCCGTGATGTAGGGTAA3’ |
|  | R: 5’TGGGTGGTGGAGTGAGAATAAG3’ |
| ZIC2 | F: 5’GCACATTGACCCATAGCACA3’ |
|  | R: 5’GACGTTTATTTTTCCCCCACA3’ |
| EGR1 | F: 5’ACGAACGCCCTTACGCTTG3’ |
|  | R: 5’GTTCATCGCTCCTGGCAAA3’ |
| SKIL | F: 5’GCCTGATGCTCCGTGTATTC3’ |
|  | R: 5’AAAGCCCCAGTGGCAAGTTC3’ |
| NF-Kβ | F: 5’GAGAGGATTTCGTTTCCGTTATG3’ |
|  | R: 5’TGTCCTTGGGTCCAGCAGTT3’ |
